# PPG and EDA dataset collected with Empatica E4 for stress assessment

**DOI:** 10.1016/j.dib.2024.110102

**Published:** 2024-01-24

**Authors:** Sara Campanella, Ayham Altaleb, Alberto Belli, Paola Pierleoni, Lorenzo Palma

**Affiliations:** Department of Information Engineering (DII), Università Politecnica delle Marche, 60131, Ancona, Italy

**Keywords:** Wearable sensors, Electronic devices, Electrodermal activity, Photoplethysmography, Stress detection

## Abstract

In response to challenging circumstances, the human body can experience marked levels of anxiety and distress. Wearable devices offer a means of real-time and ongoing data collection, facilitating personalized stress monitoring. Therefore, we collected physiological signals (blood pressure volume and electrodermal activities), using Empatica E4, from 29 subjects. A personalized protocol was developed to cause cognitive, mental, and psychological stressors since they are the ones that can be experienced in working or academic environment. We also propose a pipeline to clean and process these two signals to maximize the quality of further analysis. This study aids in the comprehension of the complex connection between stress and working situations by offering a sizable dataset made up of different physiological data. It additionally enables them to create cutting-edge stress-reduction techniques and improving professional achievement while lessening the negative impact of stress on welfare.

Specifications Table

**Table:** 

Subject	Electrical and Electronic Engineering/Applied Machine Learning/Health and medical sciences.
Specific subject area	Using a self-created protocol to replicate a realistic work environment, PPG and EDA signals were collected with the wearable device Empatica E4.
Data format	Raw, Filtered, Analysed.
Type of data	Signals, Matlab script.
Data collection	Data acquisition was performed on 29 subjects of 20 y.o. and older. It was collected at the Information Engineering Department at UnivPM from September 2022 and November 2022. All the subjects were embedded with the Empatica E4 bracelet, a wearable device that collects continuous and instantaneous physiological data through its four sensors: temperature sensor, accelerometer, EDA sensor, and PPG sensor. The subjects had to perform a protocol using several objects to simulate different actions such as Lego pieces.
Data source location	Istitution: Information Engineering Department, Università Politecnica delle Marche. City: Ancona, Marche region. Country: Italy. Latitude and Longitude: 43.58778942816075, 13.516522076954265.
Data accessibility	Repository name: EmapticaE4Stress on Mendeley Data Data identification number: DOI:10.17632/kb42z77m2g.2 Direct URL to data: https://data.mendeley.com/datasets/kb42z77m2g/2 Instructions for accessing these data: Directly from the URL
Related research article	This dataset has been used for the work published at MDPI Sensors “*A Method for Stress Detection Using Empatica E4 Bracelet and Machine-Learning Techniques*” à https://doi.org/10.3390/s23073565

## 1 Value of the Data

• Utilizing physiological signals, data was acquired from the wearable sensor, the Empatica E4 bracelet, to evaluate stress reactions.
• Different stressors are caused by our protocol to simulate a working environment such as cognitive, psychological, and emotional stressors.
• The dataset holds significant value since it consists of PPG and EDA signals recorded from 29 subjects performing a self-developed protocol with tasks of a different nature.
• The dataset can be used to refine the classification of stress levels based on physiological signals collected from wearable device, analyze physiological signals, train and test AI models, and validate findings.

## 2 Background

Stress is one of the primary causes of both physical and mental disorders in humans It serves as the body's coping mechanism in difficult or unpleasant situations, and it never stops trying to restore equilibrium to the body [1]. Stress-related pathologies or illnesses are thought to be the second most common cause of sickness in both Europe and the US, accounting for three out of every four medical visits [2]. This dataset has been collected to study if there is a correlation between different tasks, how long it takes to recover from a stress situation in a working environment and how to handle it. Moreover, we want to make available labelled and original signal datasets for use in analysis, classification, and prediction in related future studies. In general, the goal of this research is to advance our knowledge of how stress impacts performance and open new avenues for future research in this field.

## 3 Data Description

The proposed data repository is organized as follows: a folder called “Subjects” in which there are 29 folders, one for each subject named “subject_xx” where xx is an increasing number ID. In each folder, there are six .csv file with all the data recorded by Empatica E4. In Table 1 it is reported a complete description of each of them.

**Table 1:** Description of the signals.

Data	Data description	File name
Blood Volume Pressure (BVP)	Participant's blood volume pressure collected by the photoplethysmography (PPG)	BVP.csv
Electrodermal Activity (EDA)	Participant's electrodermal activity	EDA.csv
Body Temperature (TEMP)	Participant's body temperature, measured in °C.	TEMP.csv
Heart rate (HR)	Participant's 10s-average heart rate	HR.csv
Inter-beat interval (IBI)	The time interval between individual beats	IBI.csv
Accelerometer data (ACC)	Participant's 3-axis accelerometer value	ACC.csv

In the supplementary materials, two MATLAB files are stored: “EDA.m” and “PPG.m”. They have been used to preprocess and analyze only the PPG and EDA acquisition. In Table 2, a deeper explanation of both scripts is provided.

**Table 2:** Description of the Matlab scripts.

Matlab file	File description
PPG.m	In this MATLAB file, firstly we split the data into different segments since there is one task without a fixed duration. After that, we add the labels (0 for rest and 1 for stress). Finally, we performed the segments rejection and then the filtering.
EDA.m	In this MATLAB file, we resample the signal, split the data into different segments since there is one task without a fixed duration. After that, we add the labels (0 for rest and 1 for stress) and perform the filtering.

## 4 Experimental Design, Materials and Methods

### 4.1 Device

This study made use of the Empatica E4 bracelet, a wearable device, featuring four sensors - an accelerometer, PPG, EDA, and temperature- which collects physiological data continuously in real time. To guarantee stability during testing, the E4 was securely fastened around the wrist. The E4 Realtime app on a smartphone was used to monitor data in real time and store it through Bluetooth streaming acquisition. After that, the data were uploaded to E4 Connect, Empatica's cloud platform, for preliminary processing. The session can be viewed directly on the website or downloaded as a zip file containing multiple signals, including temperature, heart rate, acceleration, BVP (PPG), electrodermal activity (EDA), and interbeat intervals (IBI). Only EDA and BVP signals have been examined for this study. They were gathered from various sensors that have the following characteristics: **PPG Sensor**: This sensor samples at a rate of 64 Hz and utilizes 4 light-emitting diodes (2 green and 2 red) along with 2 photodiodes to capture signals. Green light provides information on heartbeats, while red light helps reduce motion artifacts.

• **EDA Sensor**: The Electrodermal Activity (EDA) sensor measures skin electrical conductance changes at a rate of 4 Hz, within the range of 0.01 to 100 µS, with a resolution of 900 pS. It uses stainless steel (standard), or Silver (Ag) plated with a metallic core, electrodes placed on the wrist, and applies a small alternating current to the skin.
• **Temperature Sensor**: Using an optical infrared thermopile, the TEMP signals (sampling frequency: 4 Hz, resolution: 0.02 °C) were captured and stored in the TEMP.csv file.
• **Accelerometer Sensor**: A three-axis accelerometer that measures acceleration on the x, y, and z axes within the ± 2 g range is used, with a sampling frequency of 32 Hz and resolution of 8 bits.

### 4.2 Study population

The data have been collected in our laboratory at Information Engineering Department (Università Politecnica delle Marche), after the candidates have been instructed about the protocol, the aim of this study, and signed privacy questionnaires. A total of 29 subjects (21 male and 8 female) from 20 to 60 years of age have been enrolled for the study to ensure variety.

### 4.3 Protocol description

**Fig. 1:**
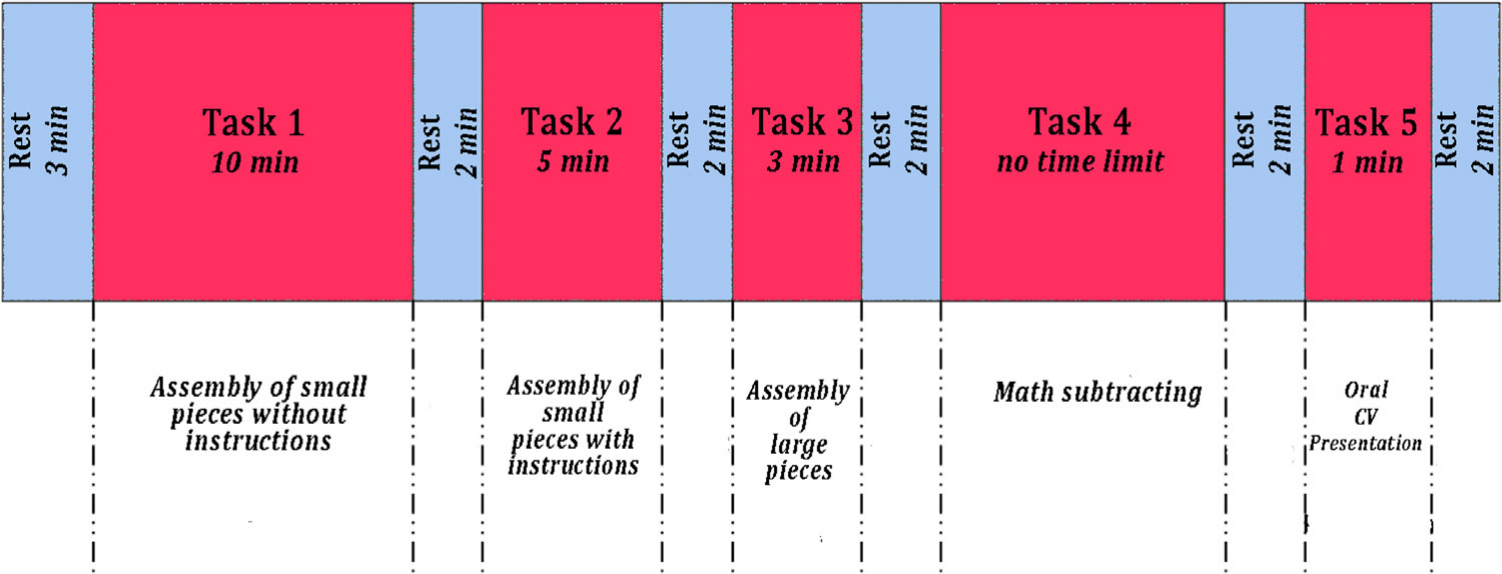
Data acquisition protocol: the blue blocks represent the rest segments while the red ones report the five tasks and their duration.

We conducted a literature search based on the various stressors to develop a protocol that integrated different stressors to produce more realistic mental stress. Since cognitive load is easily induced in a laboratory setting and is most likely to be activated in a work context, we concentrated primarily on producing cognitive, social, and physiological stressors. Consequently, we developed the protocol that is depicted in Fig. 1, which was adapted from the one suggested in [3].

To create a baseline, the bracelet was turned on and three minutes of rest were recorded. There was a two-minute break after every activity. In the first task, participants had 10 min to put together a Lego object with no instructions—just the pictures printed on the box while the second assignment consisted in assembling the identical Lego construct in five minutes with instructions. In the third activity, participants must follow instructions and count backwards from 180 (the total amount of time allocated to complete the assignment) to zero to create another Lego creation comprised of larger pieces. Each of the previously described tasks was created to mimic manufacturing processes like assembly and manual handling as well as to create the mental strain that employees may experience when performing a certain job. The fourth test is all mathematics and consists of subtracting 13 from 511 backwards repeatedly. This task does not have a time constraint. The paradigm for this test was the Montreal Imaging Stress test, which was created to investigate the impact of psycho-social stress on the human brain. [4]. Since it has been shown that oral presentations can lead to stress and memory problems, the fifth and final test requires the participant to deliver a one-minute oral presentation about themselves and their CV [5].

### 4.4 Data pre-processing

**Fig. 2:**
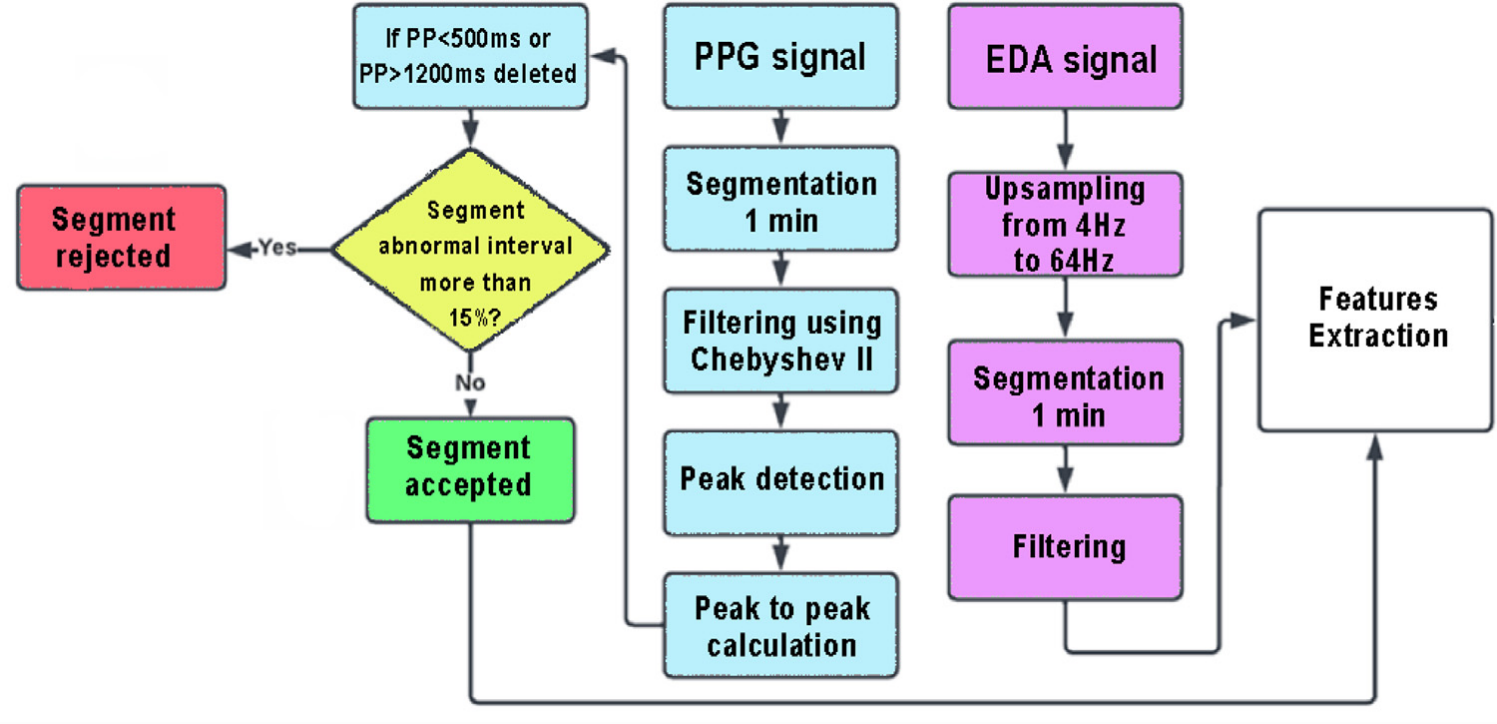
EDA and PPG pre-processing steps. There are two flowcharts: the light blue one represents the PPG pre-processing steps while the yellow one shows the decision process to discard some segments; instead, the purple one represents the different phases to clean the EDA signals.

All the steps have been performed on Matlab 2022b and they are reported in Fig. 2 and have been performed only for the PPG and EDA signals.

A pre-processing methodology was employed that initiated the segmentation of the PPG and EDA signals into intervals of 1-minute duration.

Regarding the PPG signals, numerous noises and artefacts might alter the signals during PPG recording, decreasing the stress-detection system's accuracy. The motion artefact is the most common one and has a major effect on the quality of the PPG signal. For this reason, all the segments were filtered using a Chebyshev II order-4 filter with a stopband attenuation of 20 dB and a passband of 0.5–5 Hz [6]. Peak detection was performed using the *findpeaks* function, with a threshold set to a minimum peak distance of 0.4 s and a minimum peak height of 0. Afterwards, peak-to-peak matrices were calculated by subtracting every two consecutive peaks. Therefore, only intervals with a time duration of 500 to 1200 ms (corresponding to heart rates of 120 and 50 beats per minute) were taken into consideration, while all abnormal intervals (time duration less than 500 ms or greater than 1200 ms) were excluded. PPG segments with abnormal intervals that made up less than 15 % of all intervals were therefore considered. The threshold of 15 % was selected to ensure that the selected PPG segment still has a time length greater than the 50 s after removing abnormal intervals [7].

Upsampling from 4 to 64 Hz was necessary for EDA pre-processing to get both signals at the same sampling frequency. To remove any artifacts, smoothing using the Gaussian low pass filter, with a 40-point window and sigma of 400 ms, was carried out [8].

The processed signals can now be used to further analysis or features extraction process.

## Limitations

The limitations of the dataset stem from the inadequate rest period between tasks and the motion artifacts resulting from the required protocol movements, contributing to significant noise. The short intervals for rest can affect the transition from stress to rest and, therefore, the subjects are not completely relaxed. Motion artifacts, due to the different movements performed during the protocol, introduce substantial noise to the data. These artifacts can distort recordings, compromising the integrity of gathered information and complicating subsequent analyses.

## Ethics Statement

Ethics review and approval were waived for this study because the retrospective analysis of the recorded data was conducted using completely anonymous data. The experimental study did not involve any invasive or medical procedures and introduced no lifestyle changes. All subjects gave their informed consent before the collection and acquisition of the data, which was carried out in compliance with the ethical principles of the Helsinki Declaration.

## CRediT authorship contribution statement

**Sara Campanella:** Data curation, Visualization, Methodology, Investigation, Writing – original draft, Writing – review & editing. **Ayham Altaleb:** Data curation, Investigation, Methodology, Writing – review & editing. **Alberto Belli:** Supervision, Conceptualization, Investigation. **Paola Pierleoni:** Supervision, Conceptualization. **Lorenzo Palma:** Supervision, Methodology, Investigation, Writing – review & editing.

## Data Availability

EmpaticaE4Stress (Original data) (Mendeley Data)
